# Finland’s handling of selenium is a model in these times of coronavirus infections

**DOI:** 10.1017/S0007114520003827

**Published:** 2020-09-29

**Authors:** Jan Ulfberg, Romana Stehlik

**Affiliations:** 1 Circad Health, 713 31 Nora, Sweden, email jan.ulfberg@circad.se; 2 Department of Surgical Sciences, Uppsala University Hospital, 752 37 Uppsala, Sweden

The very recent article by Bermano *et al.* highlights the importance of Se status in Covid-19 infections^(1)^. Further articles recently published also show the ability of Se to prevent, forestall and/or alleviate the clinical course of Covid-19 infection^(2–5)^.

The major difference between countries and regions regarding death rates in Covid-19 is significant. However, there are major differences between countries in terms of diagnosis of the disease – among other things, the frequency of autopsies varies. The propensity of reporting probably also varies among various countries.

Several European countries have low Se levels in their soils^(6)^. Two neighbouring countries in northern Europe have particularly low values in this context, namely Sweden and Finland^(7,8)^.

Finns, however, now derive enough Se from food, as agricultural fertilisers are supplemented with Se, a measure which Finland was the first country in the world to introduce as early as 1984. It turned out that, after the introduction of Se fertiliser, the level of Se in the blood of the Finnish population increased by about 55 % and the Finnish level is thus high by international standards^(9)^. Sweden has not supplemented the agricultural fertilisers with Se, and the current Se in blood in Swedes is not known.

The use of Se fertiliser is considered to be the safest way to ensure an adequate Se content in Finnish food and feed. The fertiliser content of inorganic Se in plants is converted into organic Se compounds that humans and animals can assimilate. Se levels in food fall rapidly if cultivated plants are not supplemented with Se in the fertilisers.

Sweden has among the highest death rates in the world in Covid-19 infection. Related to the size of the population, right now in July 2020, about 5600 people, 0·0006 % of the population have died as a result of the Corona pandemic.

Finland has a completely different situation. In July 2020, in Finland alone, about 320 people, 0·00006 % of the population died of Covid-19. Sweden thus has a ten-fold greater mortality related to Covid-19 infection.

Sweden and Finland have equal access to healthcare, but the closure of society with restrictions, in order to minimise the spread of infection, has been more extensive in Finland. Regardless of different measures to reduce the spread of infection in these two neighbouring countries, the difference in mortality in disease caused by Covid-19 is striking. One hypothesis may therefore be that different Se status of the population living in different countries or regions is important in this context^(4,5)^.

Kieliszek & Lipinski^(10)^ have recently argued for the use of a Se salt, sodium selenite, in Covid-19 infection. Sodium selenite is said to be able to inactivate one of the virus’s proteins, so that the virus is prevented from penetrating into healthy cells. The virus is thus rendered harmless^(10)^. However, there are no placebo-controlled clinical trials on virus treatment with Se tablets. Incentives to pay for studies on the effect of an element such as Se for the purpose of treating a viral infection do not exist on the part of business. Patents on an element are of course lacking. If we wish to see this type of study, the scientific world must take that initiative with the help of state funds.
